# A multidentate copper complex on magnetic biochar nanoparticles as a practical and recoverable nanocatalyst for the selective synthesis of tetrazole derivatives†

**DOI:** 10.1039/d4na00284a

**Published:** 2024-06-12

**Authors:** Marwan Majeed Maseer, Tavan Kikhavani, Bahman Tahmasbi

**Affiliations:** a Department of Chemical Engineering, Faculty of Engineering, Ilam University Ilam Iran t.kikhavandi@ilam.ac.ir; b Department of Chemistry, Faculty of Science, Ilam University P. O. Box 69315516 Ilam Iran b.tahmasbi@ilam.ac.ir bah.tahmasbi@gmail.com

## Abstract

Waste recycling, novel and easy methods of recycling catalysts, use of green solvents, use of selective catalysts and preventing the production of by-products are the most important principles of green chemistry and modern technology. Therefore, in this work, biochar nanoparticles (B-NPs) were synthesized by the pyrolysis of chicken manure as a novel method for waste recycling. Subsequently, the B-NPs were magnetized by Fe(0) nanoparticles to improve the easy recovery of biochar. Then, the surface of biochar magnetic nanoparticles (FeB-MNPs) was modified by (3-chloropropyl)trimethoxysilane (3Cl-PTMS). Finally, a multidentate copper complex of 2,2′-(propane-1,3-diylbis(oxy))dianiline (P.bis(OA)) was immobilized on the surface of modified FeB-MNPs, which was labeled as Cu-P.bis(OA)@FeB-MNPs. Cu-P.bis(OA)@FeB-MNPs was investigated as a commercial, homoselective, practical, and recyclable nanocatalyst in the synthesis of 5-substituted-1*H*-tetrazole compounds through the [3 + 2] cycloaddition of sodium azide (NaN_3_) and organo-nitriles in polyethylene glycol 400 (PEG-400) as a green solvent. Cu-P.bis(OA)@FeB-MNPs was characterized using wavelength dispersive X-ray (WDX) spectroscopy, scanning electron microscopy (SEM), thermogravimetric analysis (TGA), energy-dispersive X-ray spectroscopy (EDS), vibrating-sample magnetometer (VSM), atomic absorption spectroscopy (AAS) and N_2_ adsorption–desorption (Brunauer–Emmett–Teller (BET) method) techniques. Cu-P.bis(OA)@FeB-MNPs was recovered and reused for several runs in the synthesis of tetrazoles.

## Abstract 2

Waste recycling, novel and easy methods of recycling catalysts, use of green solvents, use of selective catalysts and preventing the production of by-products are the most important principles of green chemistry and modern technology.

## Graphical abstract

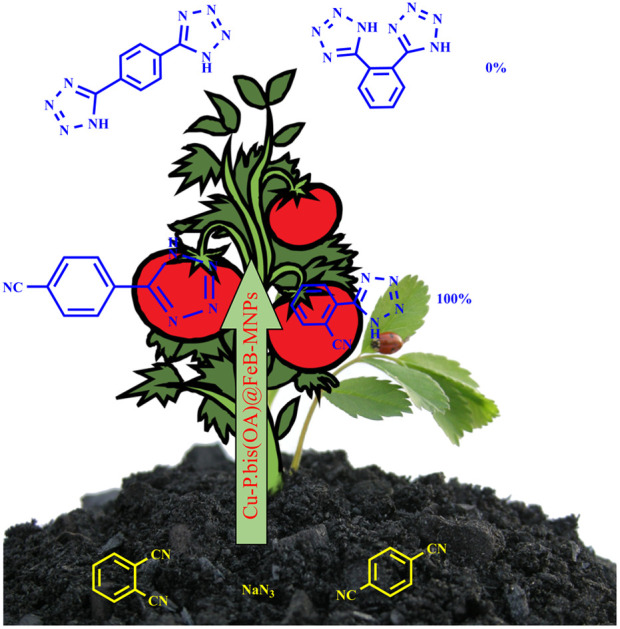

## 1. Introduction

Chemical sciences is one of the most important fields for development in the world, which provides many applications in the fields of medicine and various industries. However, unfortunately, waste chemical materials have also been introduced into the environment during the growth of chemical science. Therefore, principles of green chemistry were introduced, which minimize the environmental damage from chemical industries and laboratories. One of the principles of green chemistry is the use of recyclable catalysts.^1–5^ Acids, bases, and transition metals or metal complexes are among the most well-known types of catalysts. In general, catalysis systems are divided into two categories: homogeneous catalysis systems and heterogeneous catalysis systems.^6^ Homogeneous catalysts are known to exhibit several properties such as high performance, selectivity, instability, difficult to recover, and time-consuming product purification.^7,8^ On the other side, heterogeneous catalysts are known to exhibit different properties such as good recoverability, high stability, low efficiency, and selectivity.^7,8^ Recently, nanocatalysts have emerged as a new category of catalyst systems that act as the bridge between homogeneous and heterogeneous catalysts and have advantages over both homogeneous and heterogeneous catalysts.^6,8–12^ Because decreasing the particle size provides a high surface area, the catalytic activity and selectivity are increased (*e.g.*, homogeneous catalysts). In addition, nanomaterials have high stability and are heterogeneous in nature, enabling them to be easily recovered and reused like heterogeneous catalysts. In this context, various nanoparticles such as boehmite,^13–15^ mesoporous silica materials,^16–18^ graphene oxide,^19–21^ MOF compounds,^22–31^ carbon nanostructures,^32,33^ polymers,^34^ biochar,^35,36^ and magnetic particles^37^ have been reported as catalysts or catalyst supports. However, most of these materials are synthesized from mineral and non-renewable chemical compounds, which are against the principles of green and modern chemistry. Raw materials for biochar production include wood chips, tree bark, plant residues, animal manure, and organic wastes.^38–40^ As is known, the use of renewable materials and waste recycling are other principles of green chemistry.^41,42^ The synthesis of biochar nanoparticles from natural and renewable sources is very important in expanding its application. Considering the increasing importance of the catalysts in various industries and laboratories, the introduction of the catalysts (*e.g.* biochar) made from renewable sources is a necessity for the future. On the other hand, the increase in the population has led to the accumulation of a large amount of waste, which has become a challenge for the planet and the future of mankind. Therefore, recently, the technology of recycling waste and turning waste into valuable materials is of special interest, so that one of the principles of green chemistry is dedicated to this challenge. Considering that biochar synthesis is a novel method for waste recycling, it doubles the importance of expanding the use of biochar. Therefore, in this work, biochar nanoparticles were synthesized by the pyrolysis of chicken manure as a method for recycling agricultural waste. In fact, biochar is stable carbon black, and its surface is covered by C=O, COOH, and OH groups that provide a modifiable surface for the immobilization of catalyst species.^43–47^ Despite the special advantages of biochar, it has rarely been reported as a catalyst or catalyst support. Despite the unique advantages of biochar, the separation and recycling of biochar nanoparticles require time-consuming and difficult methods such as centrifugation and filtration. This limitation can be overcome by biochar magnetic nanocomposites as a new and ideal methodology, which can be easily separated by an external magnet.^48–51^ For example, in 2024 biochar was magnetized by magnetic Fe_3_O_4_ nanoparticles^36^ and magnetic Ni nanoparticles.^48^ But, biochar, and Fe(0) particles – with high surface area and stability – have rarely been used for the magnetization of materials.^52^ Therefore, in this work, a complex of copper on magnetic biochar nanoparticles (Cu-P.bis(OA)@FeB-MNPs) was prepared as a new recyclable nanocatalyst for the homoselective synthesis of 5-substituted-1*H*-tetrazole compounds through [3 + 2] the cycloaddition of NaN_3_ and organo-nitriles in PEG-400 as a green solvent. This is the first report on a copper complex of 2,2′-(propane-1,3-diylbis(oxy))dianiline on magnetic biochar nanoparticles and its introduction as a green catalyst in the synthesis of organic compounds.

## 2. Experimental

### 2.1. Preparation of biochar magnetic nanoparticles (FeB-MNPs)

Biochar nanoparticles were formed by simple pyrolysis of chicken manure. In this context, 500 g of dried chicken manure was heated at 400 °C as the pyrolysis temperature for 1 h under N_2_ sweeping. At the end of pyrolysis, the heating was stopped and the Chinese crucibles were cooled with N_2_ sweep. The resulting black solid was biochar. Then, 4.5 g of the synthesized biochar was mixed with FeCl_2_·4H_2_O (5.34 g) in ethanol (25 mL) and H_2_O (5 mL), and then, it was stirred at room temperature for 15 min. Then, NaBH_4_ (2.5 g in 70 mL of H_2_O) as a reduction agent was injected into the mixture dropwise within 20 min. The obtained mixture was stirred at room temperature for 15 min. The magnetic powder (biochar magnetic nanoparticles) was filtered by magnetic decantation, washed with ethanol and dried for 6 h at 90 °C.^52^

### 2.2. Modification of FeB-MNPs with (3-chloropropyl)trimethoxysilane (3Cl-PTMS)

**Scheme 1:**
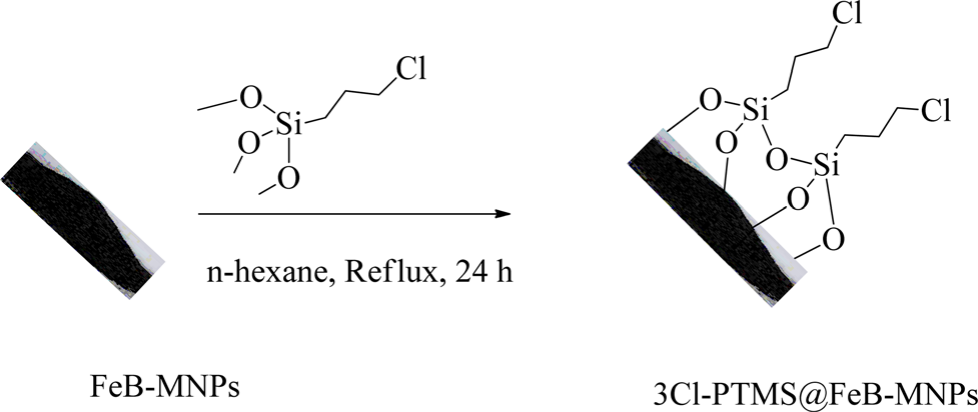
Synthesis of 3Cl-PTMS@FeB-MNPs.

FeB-MNPs (1 g) were dispersed in *n*-hexane (25 mL) for 30 min by an ultrasonic bath. Then, (3-chloropropyl)trimethoxysilane (1.5 mL) was injected into it and was allowed to stir for 24 h under reflux conditions. After 24 h, the mixture was cooled to room temperature, and the modified FeB-MNPs (3Cl-PTMS@FeB-MNPs) were separated by magnetic decantation and washed with ethanol. The modified FeB-MNPs were dried at 60 °C (Scheme 1).

### 2.3. Functionalization of FeB-MNPs with P.bis(OA) (P.bis(OA)@FeB-MNPs)

**Scheme 2:**
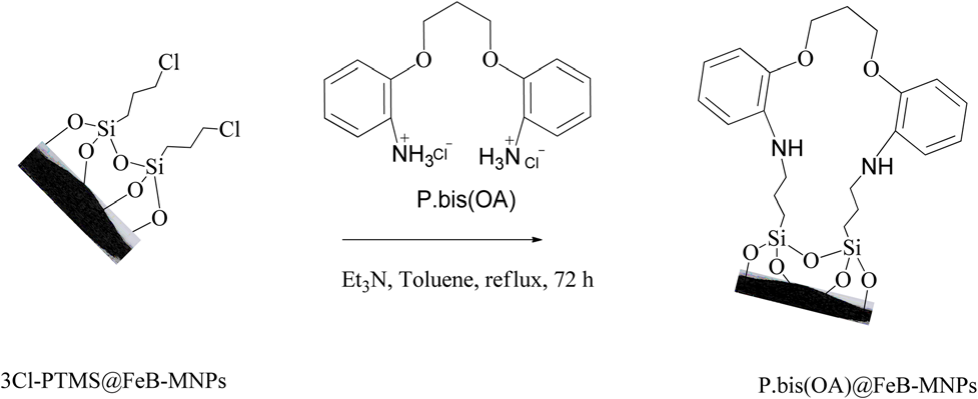
Synthesis of functionalized FeB-MNPs with P.bis(OA) (P.bis(OA)@FeB-MNPs).

FeB-MNPs (1 g) was mixed with P.bis(OA) (1 mmol) and triethylamine (8 mmol) in toluene (10 mL) and it was dispersed for 25 min in an ultrasonic bath. Then, the mixture was stirred for 72 h under reflux conditions. The mixture was allowed to cool to room temperature. After that, the functionalized FeB-MNPs with P.bis(OA) (P.bis(OA)@FeB-MNPs) were filtered by magnetic decantation and then washed 5 times with dimethyl sulfoxide (DMSO) and ethanol. The obtained P.bis(OA)@FeB-MNPs were dried at room temperature (Scheme 2).

### 2.4. Synthesis of Cu-P.bis(OA)@FeB-MNPs

**Scheme 3:**
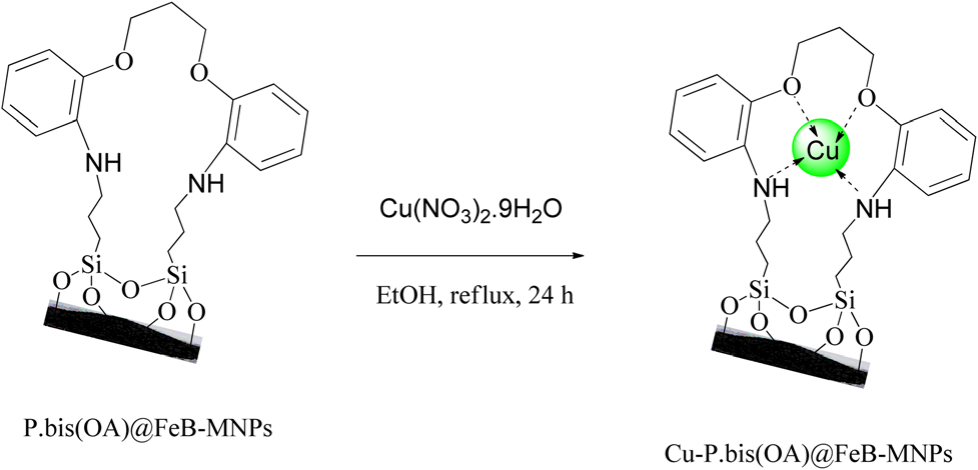
Synthesis of Cu-P.bis(OA)@FeB-MNPs.

P.bis(OA)@FeB-MNPs (1 g) was mixed with Cu(NO_3_)_2_·9H_2_O (2 mmol) in 25 mL of ethanol. The mixture was stirred under reflux conditions for 24 h. After that, the mixture was cooled. Then, the prepared final catalyst (Cu-P.bis(OA)@FeB-MNPs) was separated by magnetic decantation and washed several times with water and ethanol to remove excess copper from the mixture. Finally, Cu-P.bis(OA)@FeB-MNPs were kept at a temperature of 50 °C, which was dried (Scheme 3).

### 2.5. Synthesis of tetrazoles catalyzed by Cu-P.bis(OA)@FeB-MNPs

**Scheme 4:**
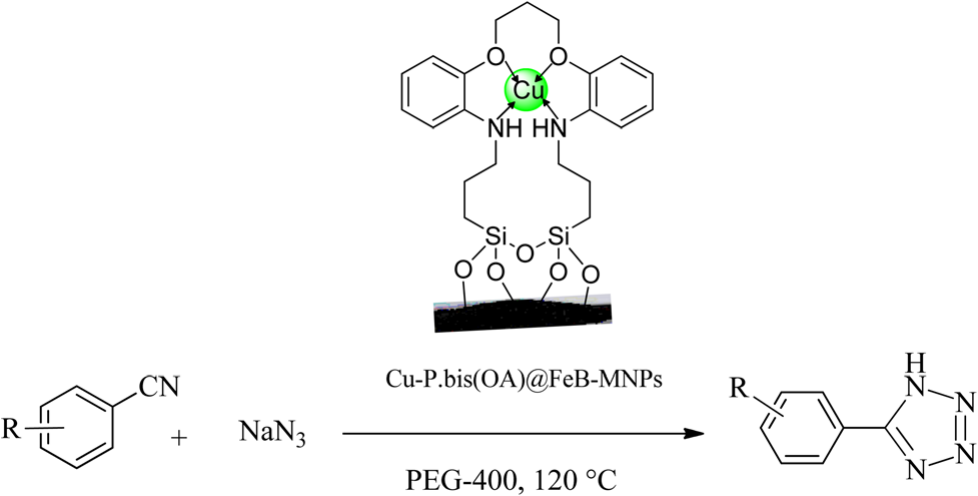
Synthesis of tetrazoles in the presence of Cu-P.bis(OA)@FeB-MNPs.

The catalytic application of Cu-P.bis(OA)@FeB-MNPs was investigated in the synthesis of tetrazoles through a ring addition reaction of nitrile and sodium azide (Scheme 4). A mixture of nitrile (1 mmol), sodium azide (1.4 mmol) in PEG-400 solvent, and 30 mg of Cu-P.bis(OA)@FeB-MNPs was stirred at a temperature of 120 °C. The reaction time was monitored by thin-layer chromatography (TLC) using UV wavelengths of 254 and 356 nm in acetone : *n*-hexane (8 : 2) tank solvent. After completion of the reaction, the reaction mixture was cooled and diluted with ethyl acetate, distilled water, and HCl solution (4 N). Then, the Cu-P.bis(OA)@FeB-MNPs catalyst was isolated from the mixture and washed with ethyl acetate and HCl solution (4 N). After that, tetrazole products were extracted in ethyl acetate solvent. The organic phase was removed by evaporation to obtain 5-substituted 1*H*-tetrazoles.

### 2.6. NMR spectra of the tetrazoles

#### 2-(1*H*-Tetrazol-5-yl)benzonitrile

^1^H NMR (250 MHz, DMSO): *δ*_H_ = 8.06 (d, *J* = 5.42 Hz, 2H), 7.92 (t, *J* = 7.05 Hz, 1H), 7.76 (t, *J* = 7.12 Hz, 1H) ppm.

#### 4-(1*H*-Tetrazol-5-yl)benzonitrile

^1^H NMR (250 MHz, DMSO): *δ*_H_ = 8.20 (d, *J* = 8.22 Hz, 2H), 8.08 (d, *J* = 4.95 Hz, 2H) ppm.

#### 5-(3-Nitrophenyl)-1*H*-tetrazole

^1^H NMR (250 MHz, DMSO): *δ*_H_ = 8.81 (s, 1H), 8.42 (t, *J* = 8.25 Hz, 2H), 7.88 (t, *J* = 7.62 Hz, 1H) ppm.

#### 5-Phenyl-1*H*-tetrazole

^1^H NMR (250 MHz, DMSO): *δ*_H_ = 8.04 (m, 2H), 7.59 (m, 3H) ppm.

#### 5-(2-Chlorophenyl)-1*H*-tetrazole

^1^H NMR (250 MHz, DMSO): *δ*_H_ = 7.79 (d, *J* = 5.90 Hz, 1H), 7.70 (d, *J* = 6.35 Hz, 1H), 7.58 (m, 2H) ppm.

#### 5-(4-Chlorophenyl)-1*H*-tetrazole

^1^H NMR (250 MHz, DMSO): *δ*_H_ = 8.03 (d, *J* = 6.65 Hz, 2H), 7.67 (d, *J* = 6.75 Hz, 2H) ppm.

## 3. Results and discussion

In this work, a heterogeneous catalyst of copper complex was immobilized on FeB-MNPs. Then, this nanocatalyst was characterized by WDX, SEM, TGA, EDS, VSM, AAS, and BET techniques. Then, its catalytic performance was investigated in the homoselective synthesis of tetrazoles.

### 3.1. Particle size and morphological identification of Cu-P.bis(OA)@FeB-MNPs by SEM

**Fig. 1:**
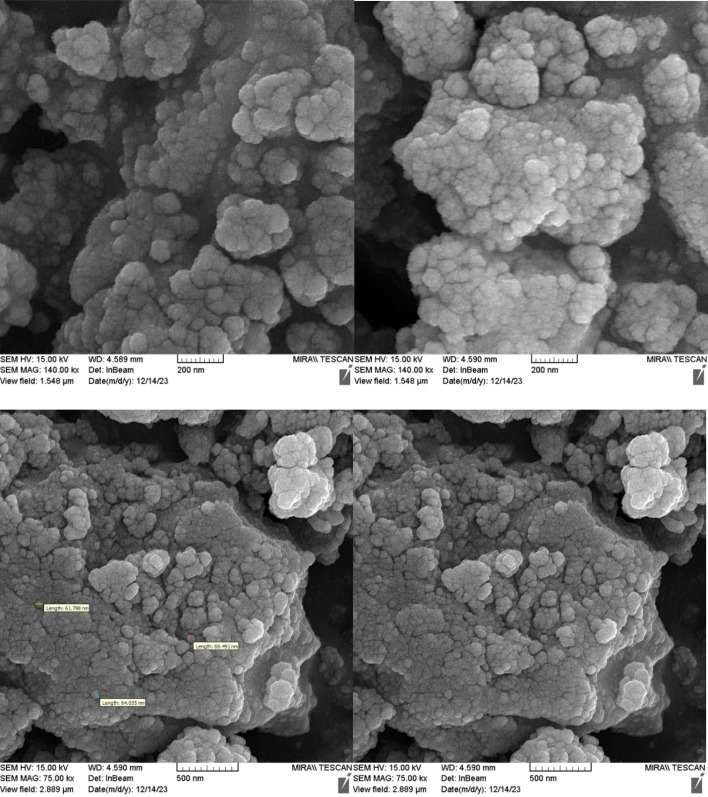
SEM images of Cu-P.bis(OA)@FeB-MNPs.

Fig. 1 shows the SEM images of Cu-P.bis(OA)@FeB-MNPs. As can be seen in SEM images, Cu-P.bis(OA)@FeB-MNPs have similar spherical shapes and uniform diameters of less than 70 nm. Also, the observed agglomeration of its particles in the SEM images is due to the magnetic nature of these nanoparticles.

### 3.2. Qualitative studying of the elements content of Cu-P.bis(OA)@FeB-MNPs

**Fig. 2:**
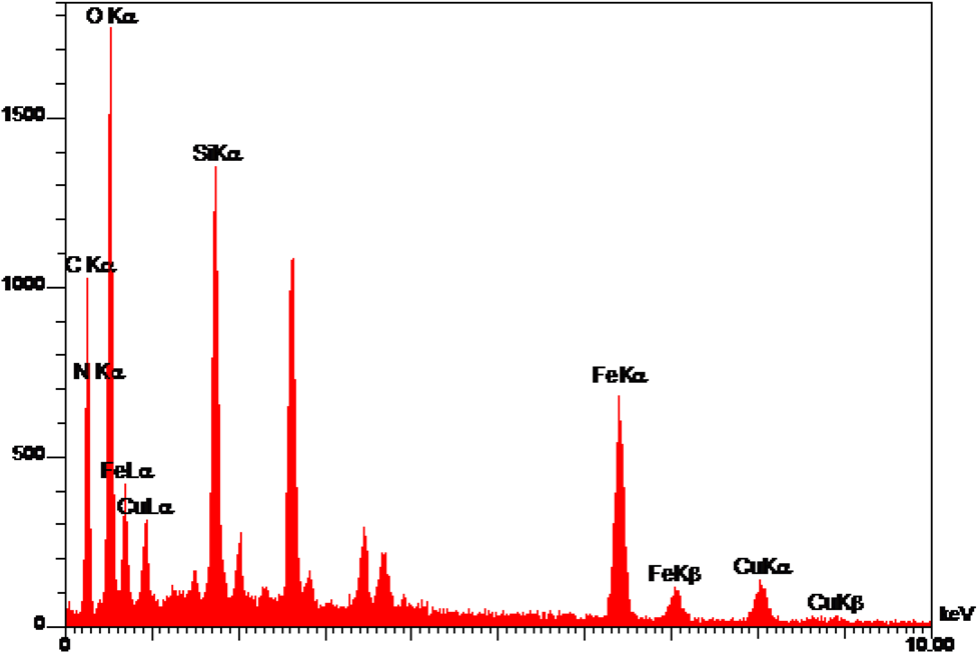
The EDS diagram of Cu-P.bis(OA)@FeB-MNPs.

EDS analysis of Cu-P.bis(OA)@FeB-MNPs is shown in Fig. 2. Magnetic biochar nanoparticles are composed of C, O, and Fe, and after their surface modification, it is functionalized with (3-chloropropyl)trimethoxysilane. Also, the 2,2′-(propane-1,3-diylbis(oxy))dianiline ligand is composed of N, O, and C, which is complexed with Cu metal. Therefore, the composition of Cu-P.bis(OA)@FeB-MNPs must include all these elements. As it is clear in the EDS analysis, all these elements are present in the composition of this catalyst, which indicates the successful synthesis of Cu-P.bis(OA)@FeB-MNPs. All C, O, Si, Cu, and N elements were observed in the catalyst structure. These evidences show that the synthesis of this catalyst was done successfully. As indicated in the EDS diagram, Cu-P.bis(OA)@FeB-MNPs are formed from C, N, O, Si, Fe, and Cu elements. The presence of Si element in this catalyst confirmed that FeB-MNPs were successfully modified by Cl-PTMS. Considering that silicon element was not present in the structure of the magnetic biochar nanoparticles and the elemental composition difference between FeB-MNPs and 3Cl-PTMS@FeB-MNPs is the presence or absence of silicon, therefore, the presence of silicon element in the elemental composition of 3Cl-PTMS@FeB-MNPs shows the successful modification of FeB-MNPs with (3-chloropropyl)trimethoxysilane. The presence of N element in Cu-P.bis(OA)@FeB-MNPs confirmed that the P.bis(OA) ligand was successfully immobilized on the surface of FeB-MNPs. Also, the presence of Cu element confirmed that copper complex was successfully synthesized on the surface of the modified FeB-MNPs.

**Fig. 3:**
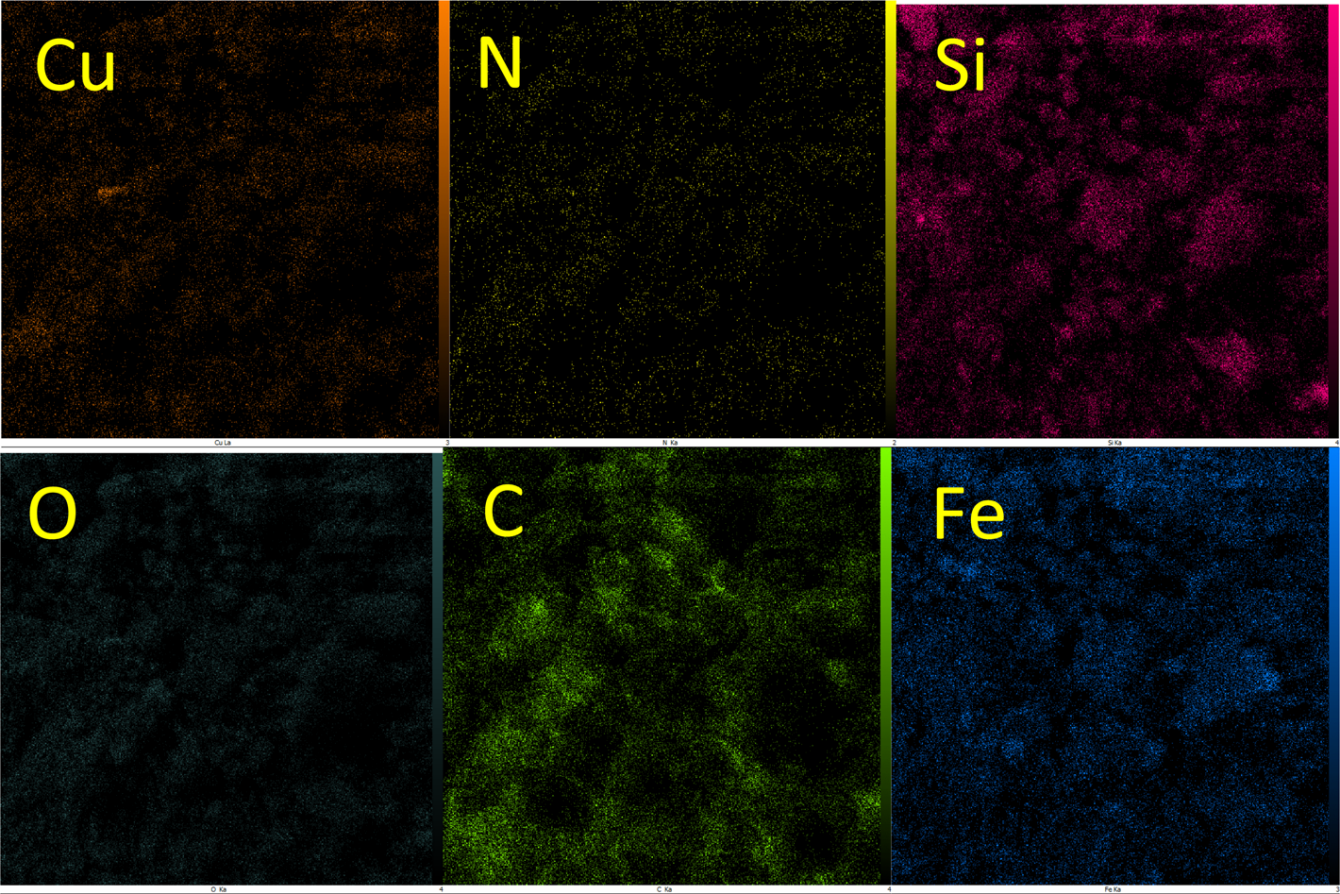
WDX images of Cu-P.bis(OA)@FeB-MNPs.

WDX analysis is another method for the qualitative study of the element content and their distribution in a sample. The WDX images of Cu-P.bis(OA)@FeB-MNPs are shown in Fig. 3. The WDX images confirmed the presence of C, N, O, Si, Fe, and Cu elements, which is in agreement with the obtained results from EDS analysis. In addition, the WDX images show a quite homogeneous distribution of C, N, O, Si, Fe, and Cu elements in the structure of Cu-P.bis(OA)@FeB-MNPs.

Because the copper element is the main catalytic active site of Cu-P.bis(OA)@FeB-MNPs, the exact concentration of Cu-metal was determined by AAS analysis, which was found to be 0.72 × 10^−3^ mol g^−1^.

### 3.3. Studying the amount of organic ligand on FeB-MNPs using TGA

**Fig. 4:**
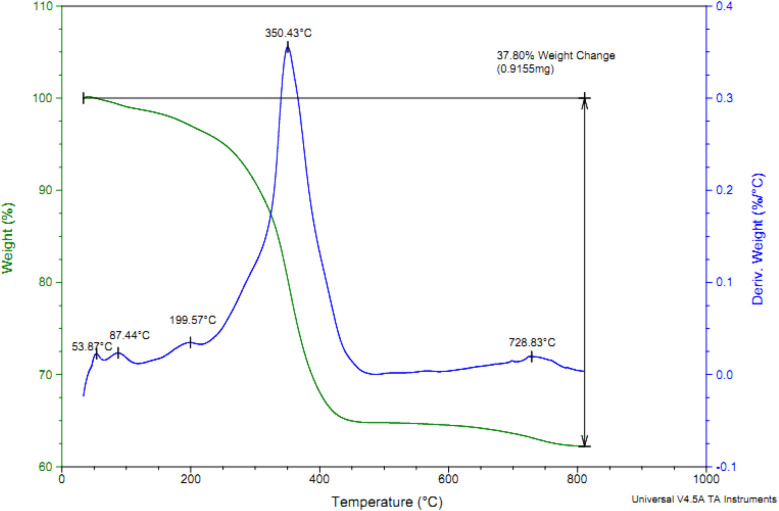
TGA/DTG diagrams for Cu-P.bis(OA)@FeB-MNPs.

The content of Cl-PTMS and P.bis(OA) as organic layers that immobilized on FeB-MNPs was studied by TGA analysis. TGA and differential thermogravimetry (DTG) diagrams of Cu-P.bis(OA)@FeB-MNPs are outlined in Fig. 4. The TGA diagram of Cu-P.bis(OA)@FeB-MNPs indicated three steps of weight loss. The first of them is due to the evaporation of the absorbent solvents, which happened below 200 °C (about 3% of weight). It is very important that no weight loss happens up to 200 °C, except for the evaporation of the absorbent solvents, which means that Cu-P.bis(OA)@FeB-MNPs is stable and applicable up to 200 °C. The immobilized organic ligands on FeB-MNPs were decomposed after 200 °C, that indicated as the second step of weight loss in the TGA diagram. Therefore, P.bis(OA) ligand was successfully immobilized on the surface of FeB-MNPs. Finally, a small weight loss above 700 °C may be due to the continuation of biochar pyrolysis.

### 3.4. N_2_ adsorption–desorption isotherms of Cu-P.bis(OA)@FeB-MNPs

**Fig. 5:**
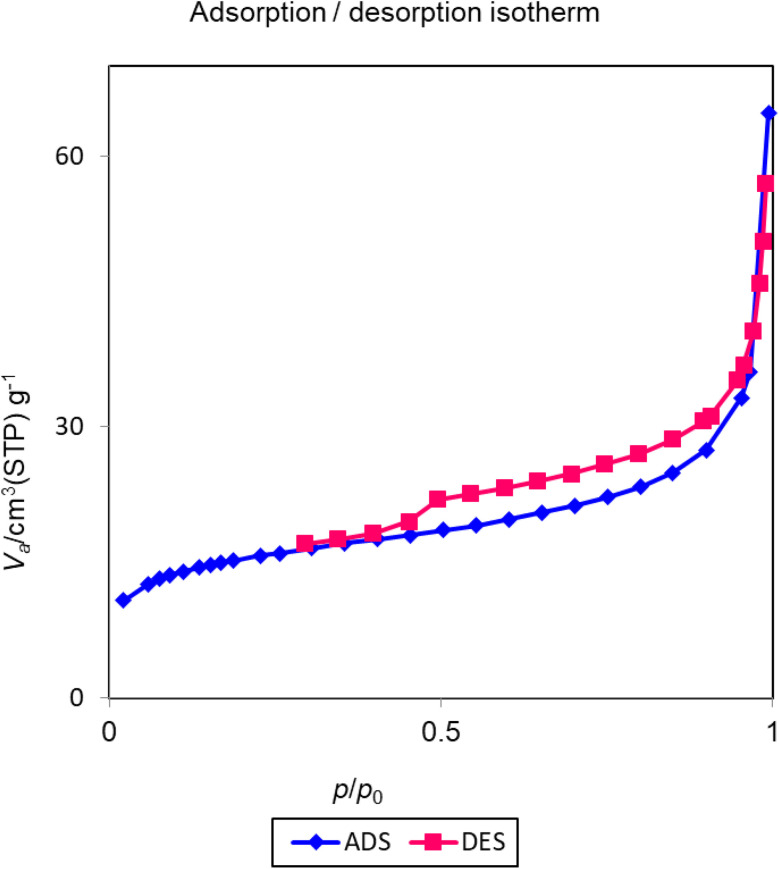
N_2_ adsorption–desorption isotherms of Cu-P.bis(OA)@FeB-MNPs.

The nitrogen adsorption/desorption technique is commonly used for the determination of the pore volume, pore diameter, and surface area of the materials. The resulting isotherms of nitrogen adsorption/desorption for Cu-P.bis(OA)@FeB-MNPs are investigated in Fig. 5. These isotherms display type H3 based on the IUPAC classification, which shows the pores structure of Cu-P.bis(OA)@FeB-MNPs.^52^ Based on BET results, the specific surface area of Cu-P.bis(OA)@FeB-MNPs is 55.23 m^2^ g^−1^. Also, the total pore volume of Cu-P.bis(OA)@FeB-MNPs is 0.09 cm^3^ g^−1^, and the average pore diameter of Cu-P.bis(OA)@FeB-MNPs is 6.81 nm.

### 3.5. VSM curve of Cu-P.bis(OA)@FeB-MNPs

**Fig. 6:**
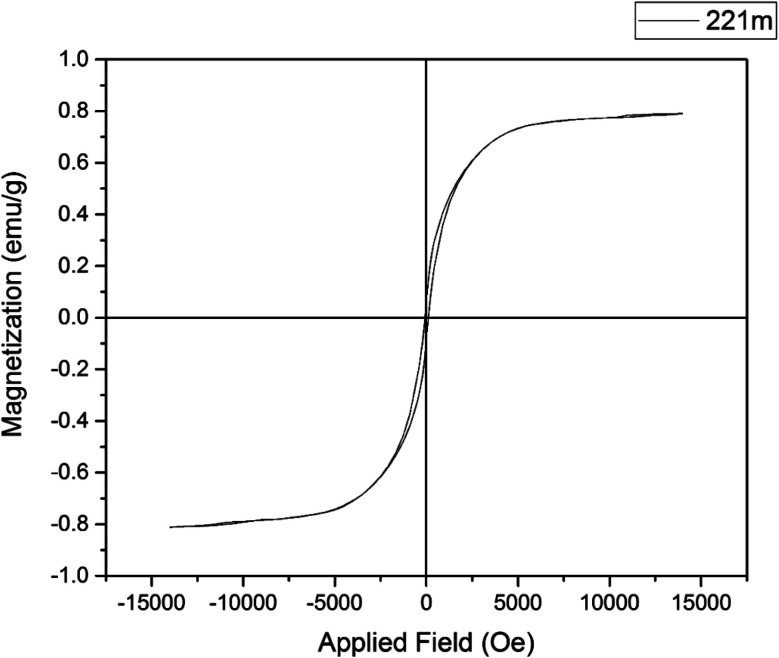
Magnetization curves for Cu-P.bis(OA)@FeB-MNPs.

The magnetic property of Cu-P.bis(OA)@FeB-MNPs was studied with VSM by the “Magnetic Kavir Kashan” device, which is outlined in Fig. 6. As shown, Cu-P.bis(OA)@FeB-MNPs showed 0.8 emu g^−1^, which is lower than the reported saturation magnetization for FeB-MNPs.^52^ The decrease in the saturation magnetic property of Cu-P.bis(OA)@FeB-MNPs is due to the grafting of Cu-complex, P.bis(OA) ligand and silica layer on FeB-MNPs.

### 3.6. Catalytic performance of Cu-P.bis(OA)@FeB-MNPs in the synthesis of tetrazoles

**Scheme 5:**
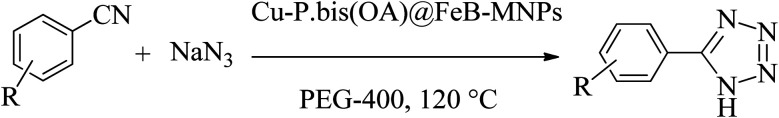
The general progress for tetrazoles synthesis catalyzed by Cu-P.bis(OA)@FeB-MNPs.

The catalytic application of Cu-P.bis(OA)@FeB-MNPs was examined for the synthesis of tetrazoles. This catalyst showed a high efficiency and good selectivity in the cyclization reaction of [2 + 3] nitriles with sodium azide in the synthesis of tetrazoles (Scheme 5).

The catalytic performance of Cu-P.bis(OA)@FeB-MNPs is described in the homoselective production of nitrogen-rich heterocyclic 5-substituted tetrazoles *via* [3 + 2]cycloaddition reaction of NaN_3_ with monofunctionalyzed benzonitriles. At first, the minimum required amount of Cu-P.bis(OA)@FeB-MNPs was checked in the [3 + 2]cycloaddition reaction of NaN_3_ with unsubstituted benzonitrile (Ph–CN) for the formation of 5-phenyl-1*H*-tetrazole. No product was formed when zero amount of Cu-P.bis(OA)@FeB-MNPs was used (Table 1, entry 1). But, when the amount of Cu-P.bis(OA)@FeB-MNPs increases, the yield of synthesized 5-phenyl-1*H*-tetrazole product increases, and the reaction time decreases. Finally, the minimum required amount of Cu-P.bis(OA)@FeB-MNPs was optimized at 30 mg (Table 1, entry 3). Next, the effect of the solvent in the synthesis of 5-phenyl-1*H*-tetrazole was checked. Volatile solvents *e.g. n*-hexane, aprotic solvents *e.g.* DMSO and protic solvents were investigated in the production of 5-phenyl-1*H*-tetrazole using 30 mg of Cu-P.bis(OA)@FeB-MNPs to obtain the optimal conditions. As clearly shown in Table 1, the production of 5-phenyl-1*H*-tetrazole increased in polar solvents with high boiling point. Finally, the best results were obtained in the PEG-400 solvent. In addition, in the study of the effect of temperature, the best results were indicated at 120 °C. The model reaction was tested on a large scale of the reactants (NaN_3_ (14 mmol, 0.9101 g) with benzonitriles (10 mmol, 1.0304 g)). The reaction yield was approximately 69% after 10 h.

**Table 1:** Optimization of reaction conditions for the production of tetrazoles in the presence of Cu-P.bis(OA)@FeB-MNPs

Entry	Catalyst (mg)	Solvent	Temp. (°C)	Time (min)	Yield^a^ (%)
1	—	PEG	120	720	NR
2	20	PEG	120	220	81
3	30	PEG	120	120	97
4	40	PEG	120	110	97
5	30	H_2_O	Reflux	360	51
6	30	EtOH	Reflux	360	58
7	30	Dioxane	Reflux	360	53
8	30	DMSO	120	360	73
9	30	*n*-Hexane	Reflux	360	20
10	30	PEG	100	180	75

a The products were separated using thin layer chromatography.

In continuation, various 5-substituted tetrazole compounds were synthesized under the above-defined conditions in the presence of Cu-P.bis(OA)@FeB-MNPs as a catalyst. The details of the experimental results such as reaction time, efficiency, and TOF values are shown in Table 2. In which, the starting material of benzonitriles having an electron-donating or withdrawing groups on *meta*, *ortho*, or *para* position of the aromatic ring were tested and successfully converted to tetrazole products. As displayed in Table 2, the final 5-substituted tetrazoles were formed in excellent yields within a fast reaction rate in the presence of Cu-P.bis(OA)@FeB-MNPs.

**Table 2:** Synthesis of 5-substituted tetrazoles catalyzed by Cu-P.bis(OA)@FeB-MNPs

Entry	Nitrile	Product	Time (min)	Yield (%)	Melting point (°C)
1	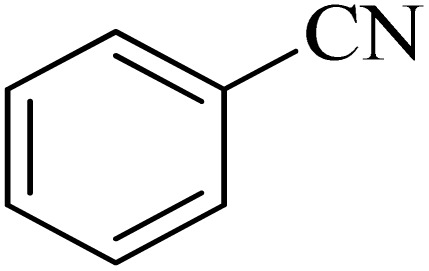	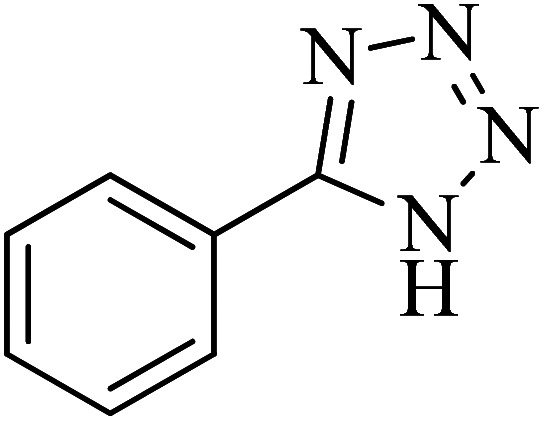	120	97	211–214 [ref. 14]
2	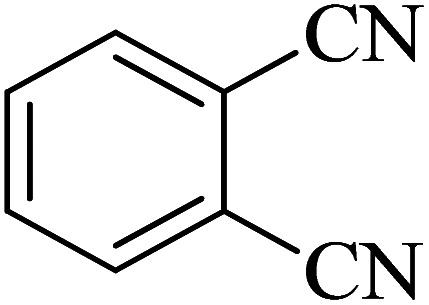	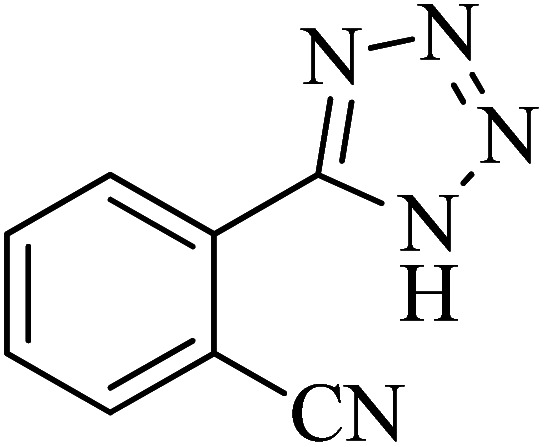	35	94	209–211 [ref. 53]
3	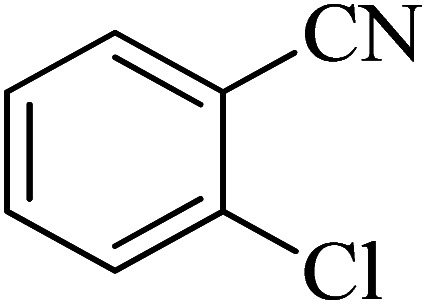	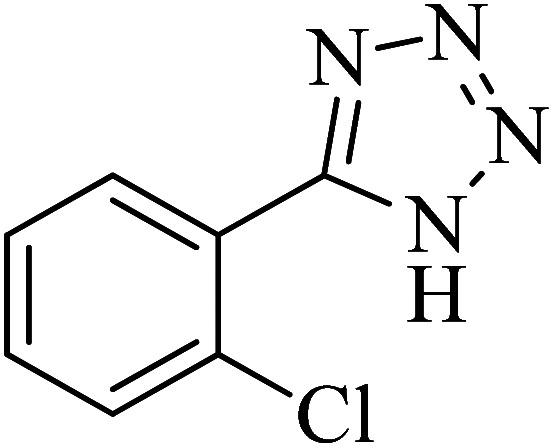	210	90	179–182 [ref. 37]
4	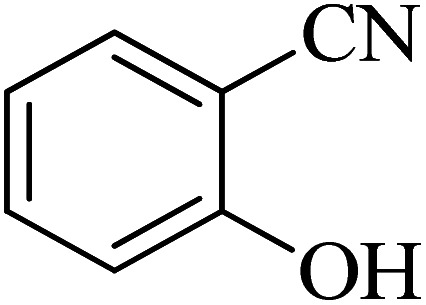	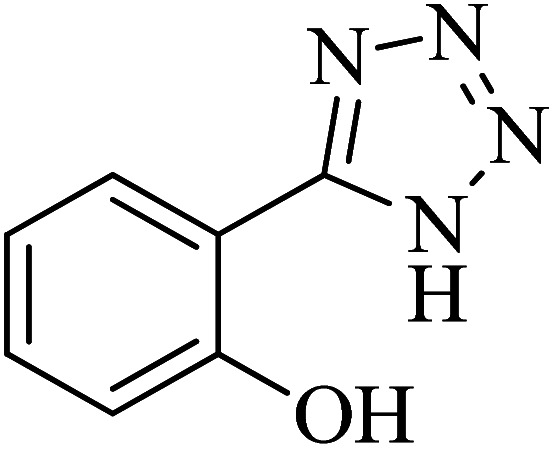	45	95	221–224 [ref. 54]
5	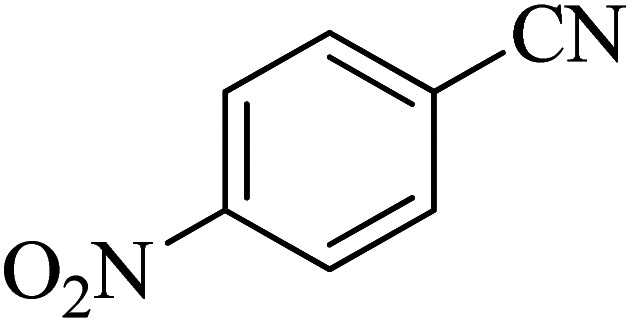	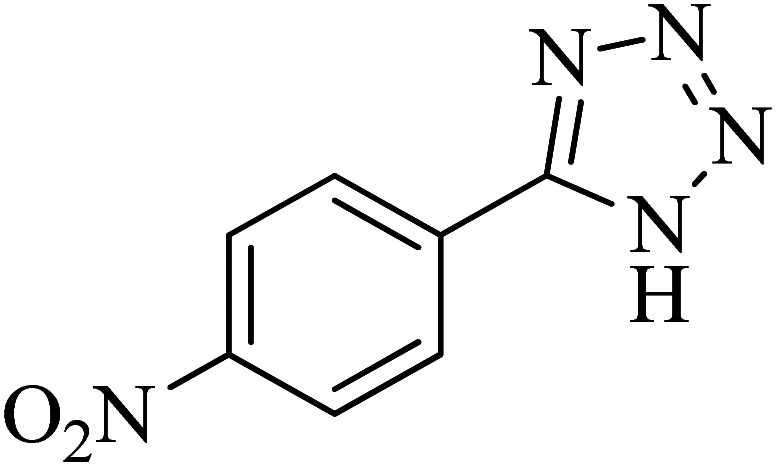	110	87	216–219 [ref. 55]
6	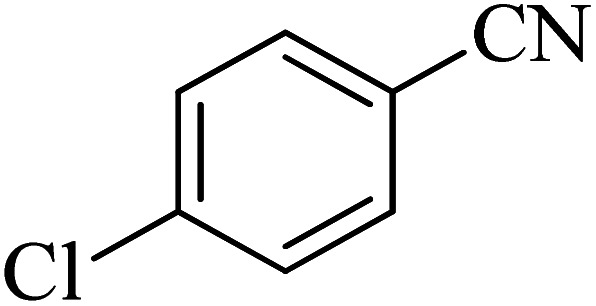	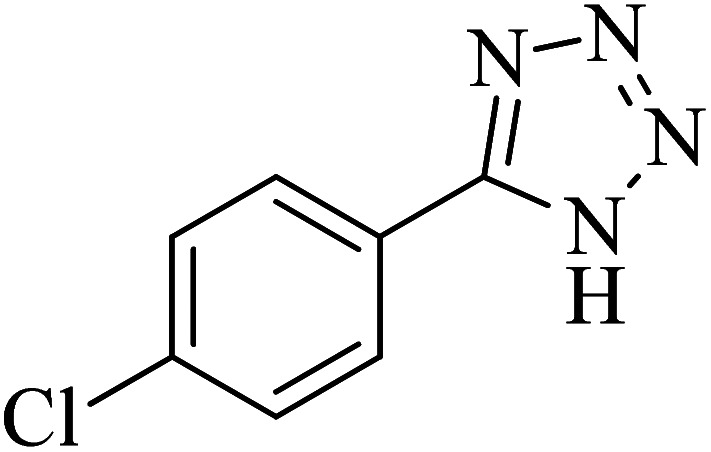	300	86	260–263 [ref. 56]
7	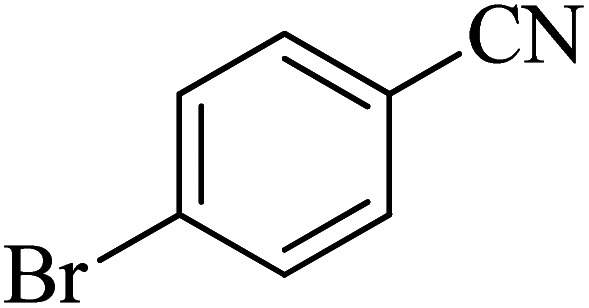	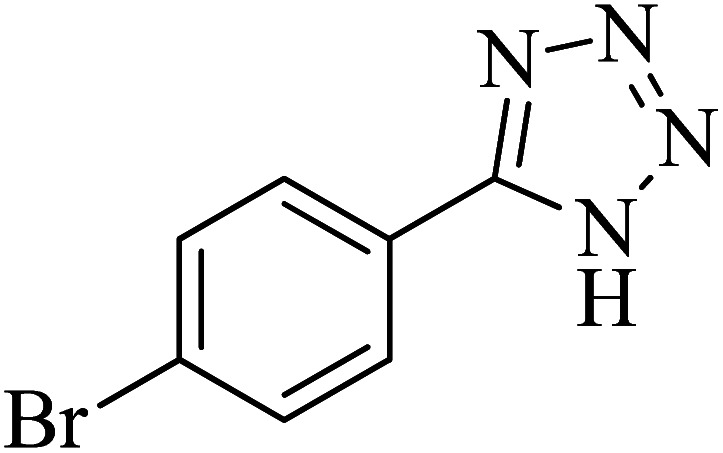	320	89	259–261 [ref. 14]
8	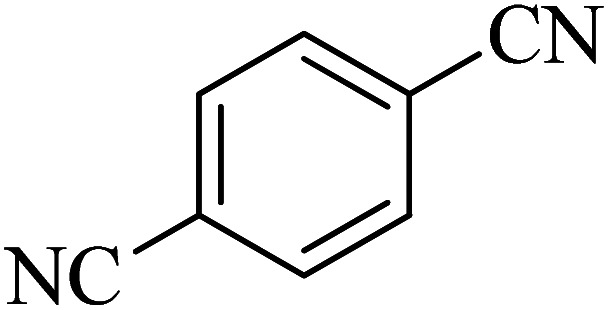	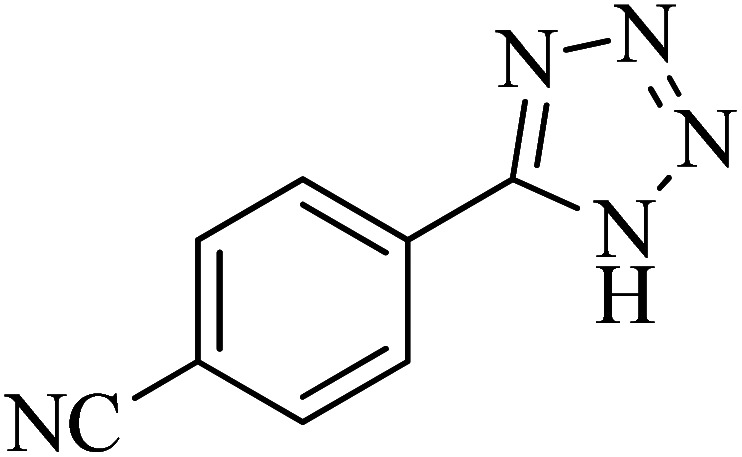	100	93	249–252 [ref. 55]
9	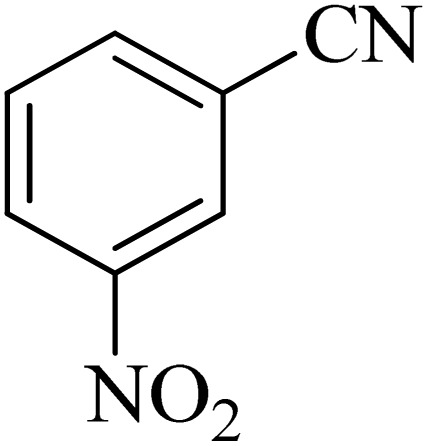	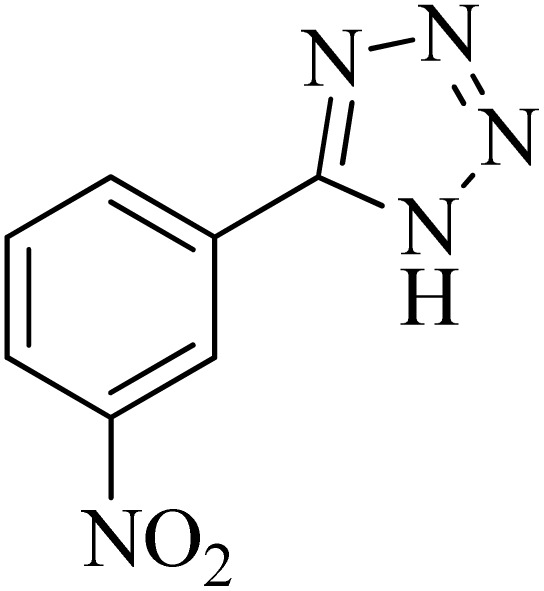	80	89	150–152 [ref. 37]

**Scheme 6:**
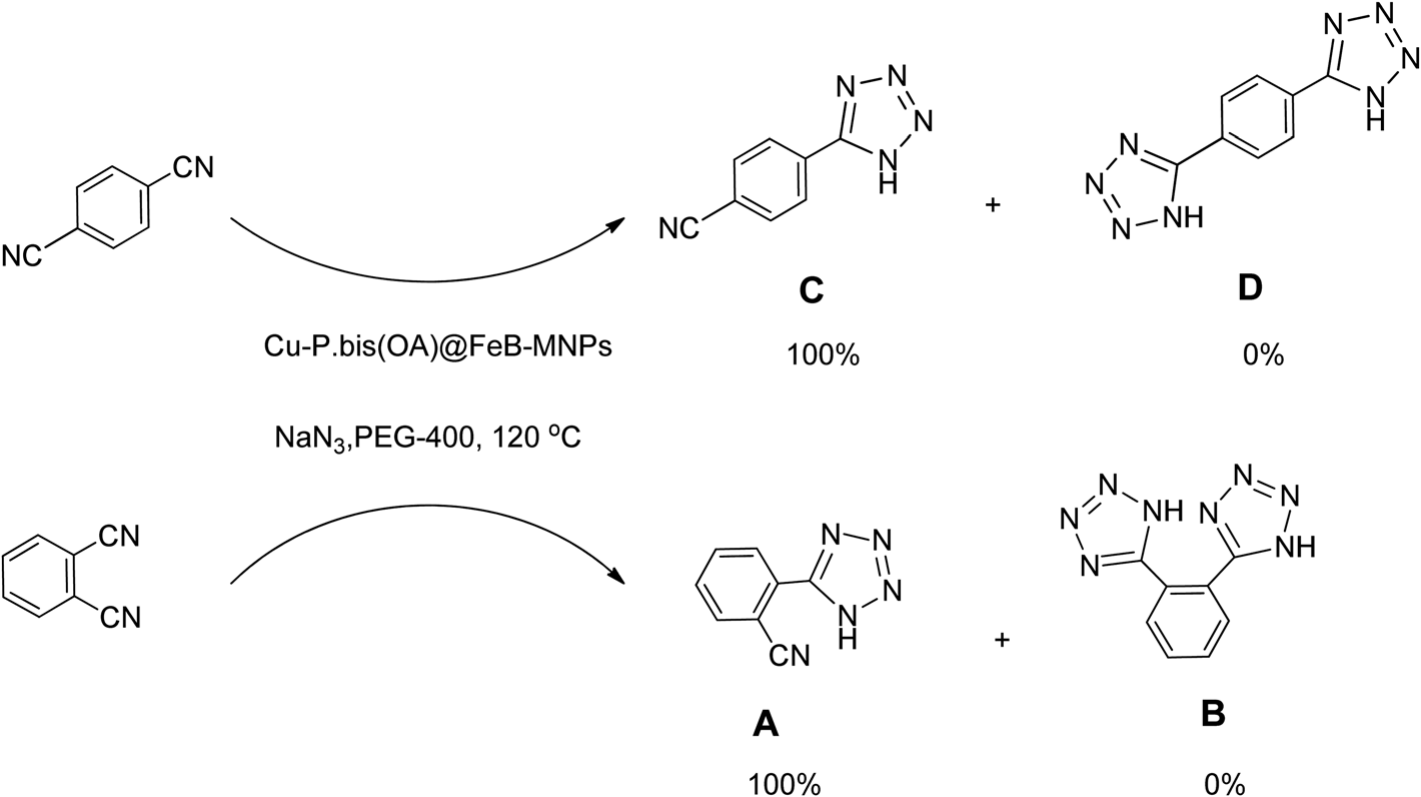
Homoselectivity of Cu-P.bis(OA)@FeB-MNPs in the [3 + 2] cycloaddition of NaN_3_ with terephthalonitrile and phthalonitrile.

**Fig. 7:**
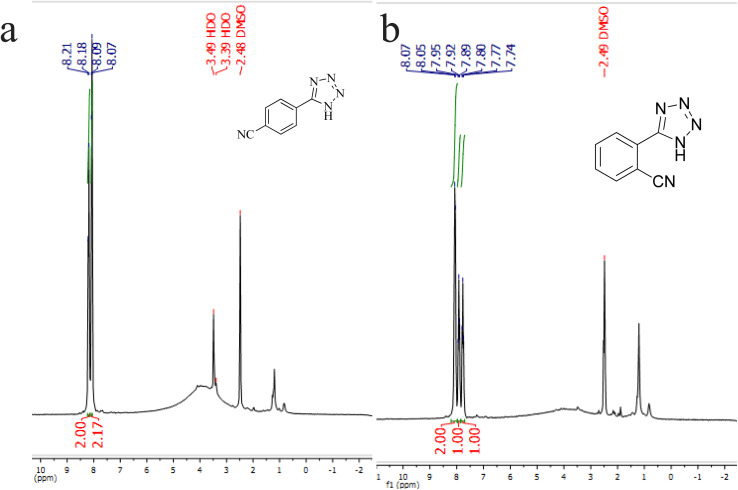
^1^H NMR spectrum of 4-(1*H*-tetrazol-5-yl)benzonitrile (a) and 2-(1*H*-tetrazol-5-yl)benzonitrile (b).

Importantly, Cu-P.bis(OA)@FeB-MNPs exhibit homoselectivity in the formation of 5-substituted tetrazoles. Because, in the reaction of NaN_3_ with dicyano-substituted benzonitriles (*e.g.* terephthalonitrile and phthalonitrile), only mono-addition happened in the presence of Cu-P.bis(OA)@FeB-MNPs (Scheme 6). These starting materials have two quite similar C≡N groups in their structure. The homoselectivity of Cu-P.bis(OA)@FeB-MNPs in the formation of 5-substituted tetrazoles through [3 + 2]cycloaddition of NaN_3_ with terephthalonitrile and phthalonitrile were investigated using ^1^H NMR. When phthalonitrile was used as the starting material, two products – 2-(1*H*-tetrazol-5-yl)benzonitrile (A) or 1,2-di(1*H*-tetrazol-5-yl)benzene (B) – were formed. As mentioned, one of the C≡N groups selectively reacted with NaN_3_ for the synthesis of 2-(1*H*-tetrazol-5-yl)benzonitrile, and the other C≡N group did not react, and the 1,2-di(1*H*-tetrazol-5-yl)benzene product was not formed. As expected, when phthalonitrile was used, if product A was synthesized, more than two peaks should be observed in the ^1^H NMR spectrum for aromatic hydrogens. But if B was synthesized, only two peaks should be observed in the ^1^H NMR spectrum for aromatic hydrogens. As shown in the ^1^H NMR spectrum of the final product from the [3 + 2] cycloaddition reaction of NaN_3_ with phthalonitrile in the presence of Cu-P.bis(OA)@FeB-MNPs, three peaks were indicated for aromatic hydrogens (Fig. 7). Therefore, only product A was certainly formed, which indicates the homoselectively of Cu-P.bis(OA)@FeB-MNPs in the synthesis of tetrazoles.

When terephthalonitrile was used as the starting material, two products – 4-(1*H*-tetrazol-5-yl)benzonitrile (C) or 1,4-di(1*H*-tetrazol-5-yl)benzene (D) were formed. As mentioned, one of the C≡N groups selectively reacted with NaN_3_ for the synthesis of C, the other C≡N group did not react, and the D product was not formed. As expected, when terephthalonitrile was used, if product C was synthesized, two peaks should have been observed in the ^1^H NMR spectrum for aromatic hydrogens. But if D was synthesized, only one peak should have been observed in the ^1^H NMR spectrum for aromatic hydrogens. As shown in the ^1^H NMR spectrum of the final product from the [3 + 2] cycloaddition reaction of NaN_3_ with terephthalonitrile in the presence of Cu-P.bis(OA)@FeB-MNPs, two peaks indicated the aromatic hydrogens (Fig. 7). Therefore, only product C was certainly formed, which indicates the homoselectively of Cu-P.bis(OA)@FeB-MNPs in the synthesis of tetrazoles.

**Scheme 7:**
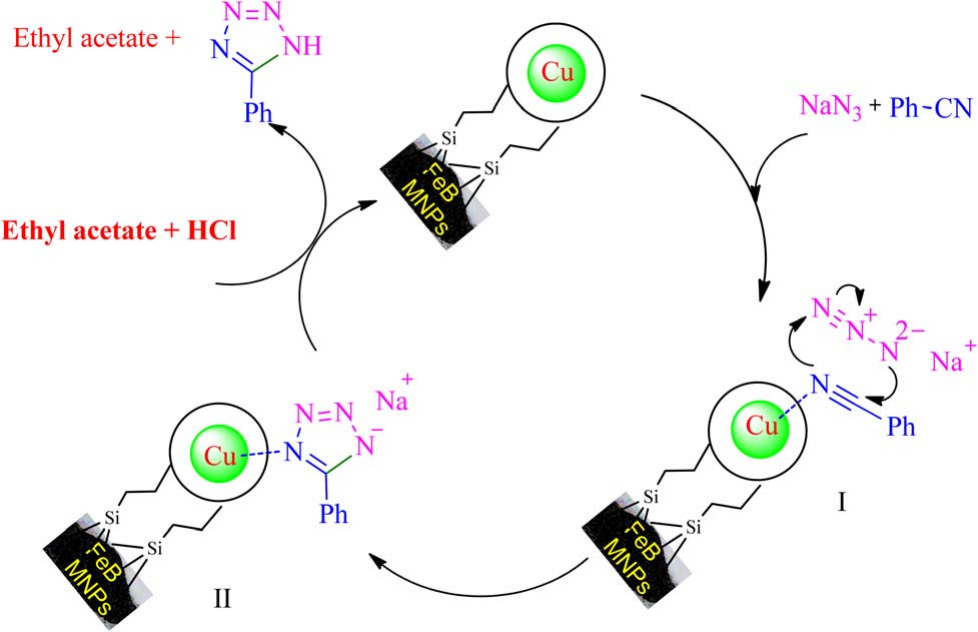
The expected catalytic mechanism for the synthesis of 5-substituted tetrazoles using Cu-P.bis(OA)@FeB-MNPs.

Based on the literature,^53,54,57^ an expected mechanism is illustrated in Scheme 7 for the synthesizing of 5-substituted tetrazoles through [3 + 2] cycloaddition reaction of NaN_3_ and nitriles in the presence of Cu-P.bis(OA)@FeB-MNPs. In this suggested mechanism, the interaction of the C≡N functional group with the active site of the catalyst causes the C≡N group to become susceptible to attack by azide ions, and the intermediate II is formed as sodium salt forms. The addition of HCl during workup converts the salt form of intermediate II to final tetrazole, which forms tetrazoles extracted in ethyl acetate.

### 3.7. Reusability of Cu-P.bis(OA)@FeB-MNPs

**Fig. 8:**
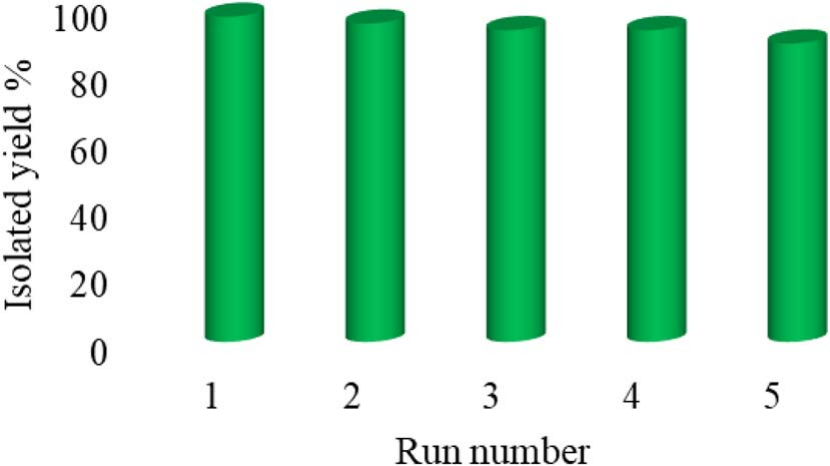
The reusability of Cu-P.bis(OA)@FeB-MNPs.

Practicability, reusability, stability, and availability are the determinative factors for catalysts. According to the emphasis of green chemistry on the reusability of catalysts, the reusability of Cu-P.bis(OA)@FeB-MNPs was investigated in the [3 + 2]cycloaddition reaction of NaN_3_ with Ph–CN toward the formation of 5-phenyl-1*H*-tetrazole. In this regard, Cu-P.bis(OA)@FeB-MNPs were isolated after the completion of the reaction and then reused again without double activation. As indicated in Fig. 8, Cu-P.bis(OA)@FeB-MNPs can be recycled up to 6 times.

**Fig. 9:**
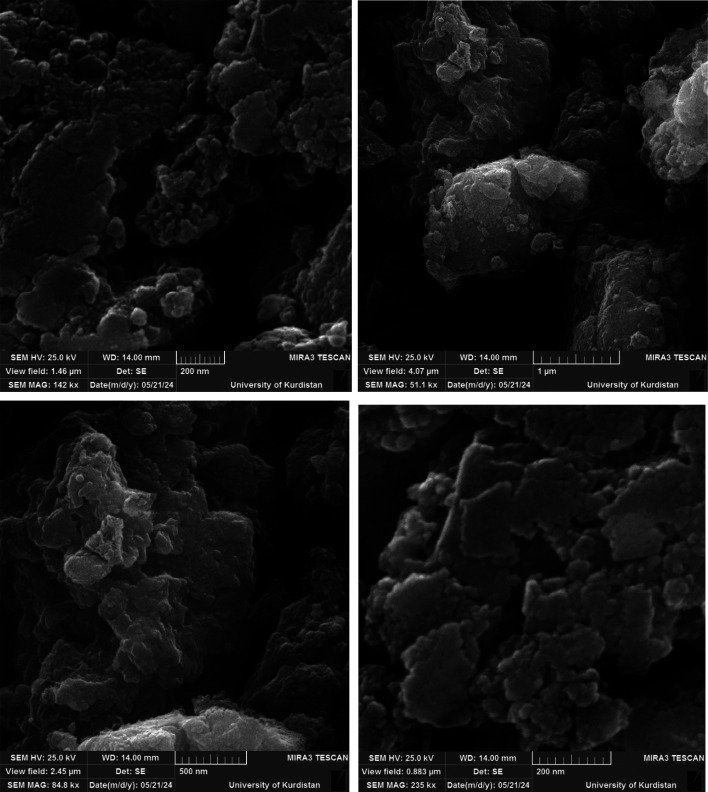
SEM images of recycled Cu-P.bis(OA)@FeB-MNPs catalyst.

Also, the recycled Cu-P.bis(OA)@FeB-MNPs nanocatalyst was characterized by SEM (Fig. 9). The SEM images of recycled Cu-P.bis(OA)@FeB-MNPs catalyst showed that the morphology and size of this catalyst did not significantly change after reusing, therefore Cu-P.bis(OA)@FeB-MNPs catalyst was stable after reusing.

### 3.8. Comparison of Cu-P.bis(OA)@FeB-MNPs with other catalysts

The practicability of Cu-P.bis(OA)@FeB-MNPs was compared with other reported catalysts (Table 3). The [3 + 2] cycloaddition reaction of NaN_3_ and Ph–CN in the presence of Cu-P.bis(OA)@FeB-MNPs was compared with other catalysts. As indicated, Cu-P.bis(OA)@FeB-MNPs exhibit 98% of the product within only 2 h, which indicates a better yield and reaction time than the other catalysts. Besides, several reports in literature formed from unrenewable materials or used hazard solvents or limited by time-consuming or difficult catalyst recovery. While FeB-MNPs are formed from renewable materials as an ideal waste recycling and Cu-P.bis(OA)@FeB-MNPs can easily be recovered and reused. More addition, 5-substituted tetrazoles were synthesized in the green solvent (PEG-400) in the presence of Cu-P.bis(OA)@FeB-MNPs.

**Table 3:** Comparison results of Cu-P.bis(OA)@FeB-MNPs with other catalysts in the formation of 5-phenyl-1*H*-tetrazole

Entry	Catalyst	Time (h)	Yield (%)	Ref.
1	CoY zeolite	14	90	58
2	Cu–Zn alloy nanopowder	10	95	59
3	B(C_6_F_5_)_3_	8	94	60
4	Fe_3_O_4_@SiO_2_/salen Cu(ii)	7	90	61
5	Fe_3_O_4_/ZnS HNSs	24	81.1	62
6	Mesoporous ZnS	36	86	63
7	AgNO_3_	5	83	64
8	CuFe_2_O_4_	12	82	65
9	Nano ZnO/Co_3_O_4_	12	90	66
10	Ni-MP(AMP)_2_@Fe-biochar	3.8	92	52
12	Fe_3_O_4_@boehmite NPs	4	97	67
13	Cu(ii)[Sal(PMeOSi)DETA]@KIT-6	3	94	68
14	KIT-6@DABP@Cu	5	91	69
15	KIT-6@SMTU@Ni	3	95	70
16	Cu-TDBB@MCM-41/Fe_3_O_4_	7	90	71
17	Nd-Schiff-base@BMNPs	3	98	48
18	Nd-bis(PYT)@boehmite NPs	2.5	97	53
19	Pd–SMTU@boehmite	2.5	95	72
20	Cu-P.bis(OA)@FeB-MNPs	2	97	This work

## 4. Conclusions

In this work, magnetic biochar NPs (FeB-MNPs) were synthesized *via* pyrolysis of chicken manure, which is a new process for recycling waste. Then, the performed biochar was magnetized by magnetic Fe(0) nanoparticles to improve its recovery. Then, a new copper complex was immobilized on its surface and used as a selective, inexpensive, stable, recoverable, practicable, and available catalyst for the synthesis of 5-substituted tetrazoles. This catalyst (Cu-P.bis(OA)@FeB-MNPs) was characterized by WDX, SEM, TGA, EDS, VSM, AAS, and N_2_ adsorption–desorption (BET method) techniques. SEM images indicated that the particles of this catalyst are less than 70 nm in size. TGA analysis confirmed that the P.bis(OA) ligand was successfully supported on FeB-MNPs and Cu-P.bis(OA)@FeB-MNPs is stable up to 200 °C. EDS and WDX confirmed that the surface of FeB-MNPs was successfully modified by Cl-PTMS and then functionalized by the P.bis(OA) ligand and the copper complex was formed on the surface of functionalized FeB-MNPs. VSM analysis indicated that this catalyst can be recovered by an external magnet. Therefore, it was recovered and reused for several runs. The BET method showed good surface area for Cu-P.bis(OA)@FeB-MNPs, therefore, this catalyst has high efficiency and activity in the synthesis of 5-substituted tetrazoles.

## Author contributions

Marwan Majeed Maseer: methodology. Tavan Kikhavani: supervision, project administration, formal analysis. Bahman Tahmasbi: supervision, conceptualization, formal analysis, resources, project administration, writing – original draft, writing review and editing.

## Conflicts of interest

The authors declare no conflict of interest and competing interests.

## Supplementary Material

NA-006-D4NA00284A-s001
