# Supplementary material for: A multidentate copper complex on magnetic biochar nanoparticles as a practical and recoverable nanocatalyst for the selective synthesis of tetrazole derivatives
Source: Nanoscale Adv. 2024 Jun 12;6(15):3948–60. doi: 10.1039/d4na00284a (PMC11265574; doi:10.1039/d4na00284a)
Supplement: NA-006-D4NA00284A-s001 [file NA-006-D4NA00284A-s001.pdf]

Electronic Supplementary Material (ESI) for Nanoscale Advances.
This journal is © The Royal Society of Chemistry 2024

Multidentate copper complex on magnetic biochar nanoparticles as a practical and recoverable nanocatalyst for the selective synthesis of tetrazole derivatives

Marwan Majeed Maseer [1], Tavan Kikhavani [1,*], Bahman Tahmasbi[2,*]

[1] *Department of Chemical Engineering, Faculty of Engineering, Ilam University, Ilam, Iran. E-mail: t.kikhavandi@ilam.ac.ir*

[2] *Department of Chemistry, Faculty of Science, Ilam University, P.O. Box 69315516, Ilam, Iran. E-mail: b.tahmasbi@ilam.ac.ir, bah.tahmasbi@gmail.com*

Abstract:

In this work, biochar nanoparticles (B-NPs) were synthesized by pyrolysis of chicken manure. Then, the B-NPs were magnetized by Fe(0) nanoparticles. Then, the surface of biochar magnetic nanoparticles (FeB-MNPs) was modified by (3-chloropropyl)trimethoxysilane (3Cl-PTMS). Finally, a multidentate copper complex of 2,2'-(propane-1,3-diylbis(oxy))dianiline (P.bis(OA)) was immobilized on the surface of modified FeB-MNPs, which labelled as Cu-P.bis(OA)@FeB-MNPs. Cu-P.bis(OA)@FeB-MNPs was investigated as commercial, homoselective, practical, and recyclable nanocatalyst in the synthesis of 5-substituted-1H-tetrazole compounds through [3+2] cycloaddition of sodium azide (NaN_3) and organo-nitriles in PEG-400 as a green solvent. Cu-P.bis(OA)@FeB-MNPs was characterized by wavelength dispersive X-ray spectroscopy (WDX), scanning electron microscopy (SEM), thermogravimetric analysis (TGA), energy-dispersive X-ray spectroscopy (EDS), vibrating-sample magnetometer (VSM), atomic absorption spectroscopy (AAS) and N_2 adsorption-desorption (BET method) techniques. Cu-P.bis(OA)@FeB-MNPs was recovered and reused for several runs in the synthesis of tetrazoles.

Keywords: Biochar nanoparticles, homoselective catalyst, magnetic nanoparticles, reusable nanocatalyst, 5-substituted-1H-tetrazoles, sodium azide, multidentate copper complex.

^{1}H NMR spectral data

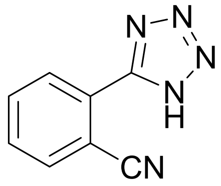

2-(1H-tetrazol-5-yl)benzonitrile

^{1}H NMR (250 MHz, DMSO): δ_H= 8.06 (d, *J*= 5.42 Hz, 2H), 7.92 (t, *J*= 7.05 Hz, 1H), 7.76 (t, *J*= 7.12 Hz, 1H) ppm.

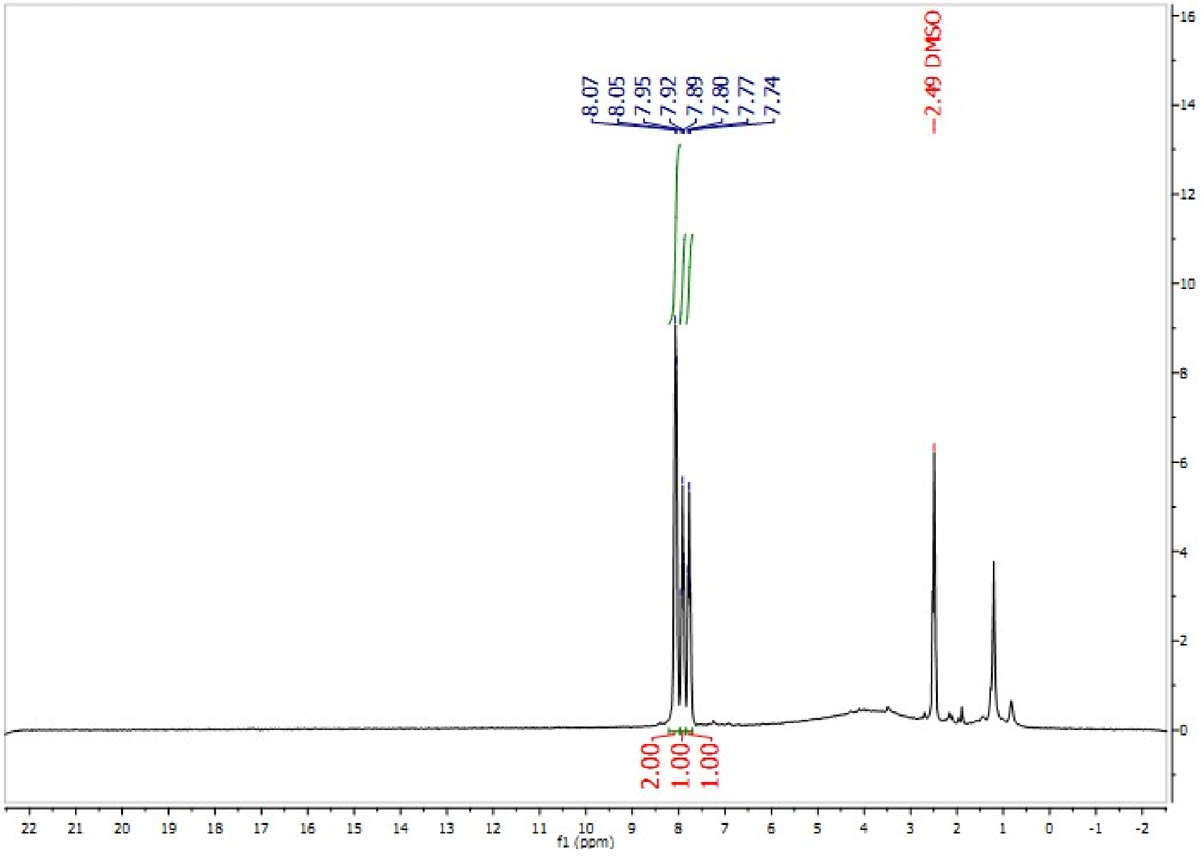

^{1}H NMR spectrum of 2-(1H-tetrazol-5-yl)benzonitrile from [3+2] cycloaddition of NaN_3 with phthalonitrile

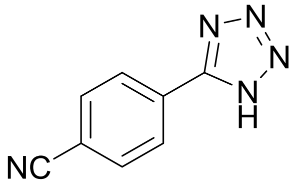

4-(1H-tetrazol-5-yl)benzonitrile

^{1}H NMR (250 MHz, DMSO): δ_H= 8.20 (d, *J*= 8.22 Hz, 2H), 8.08 (d, *J*= 4.95 Hz, 2H) ppm.

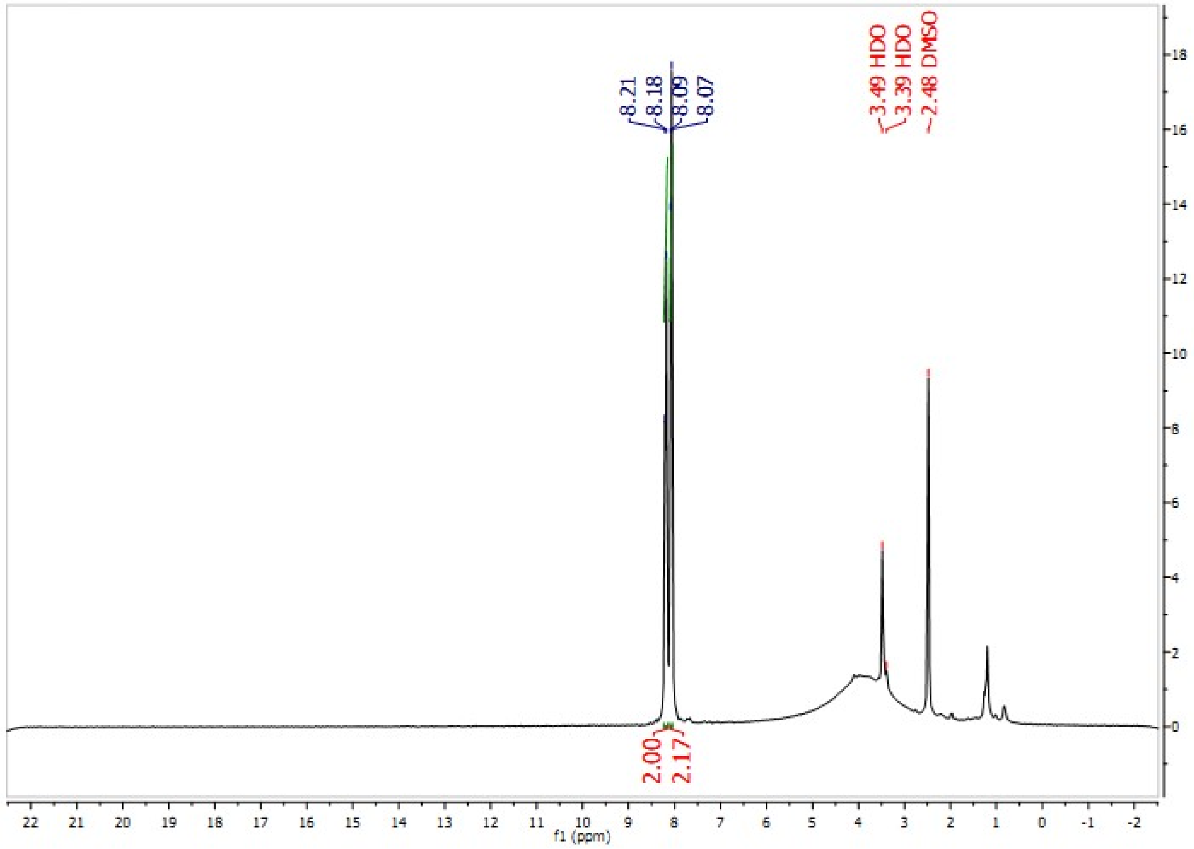

^{1}H NMR spectrum of 4-(1H-tetrazol-5-yl)benzonitrile from [3+2] cycloaddition of NaN_3 with terephthalonitrile

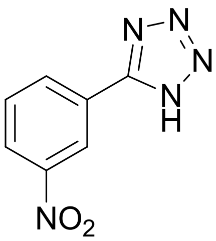

5-(3-nitrophenyl)-1H-tetrazole

^{1}H NMR (250 MHz, DMSO): δ_H= 8.81 (s, 1H), 8.42 (t, *J*= 8.25 Hz, 2H), 7.88 (t, *J*= 7.62 Hz, 1H) ppm.

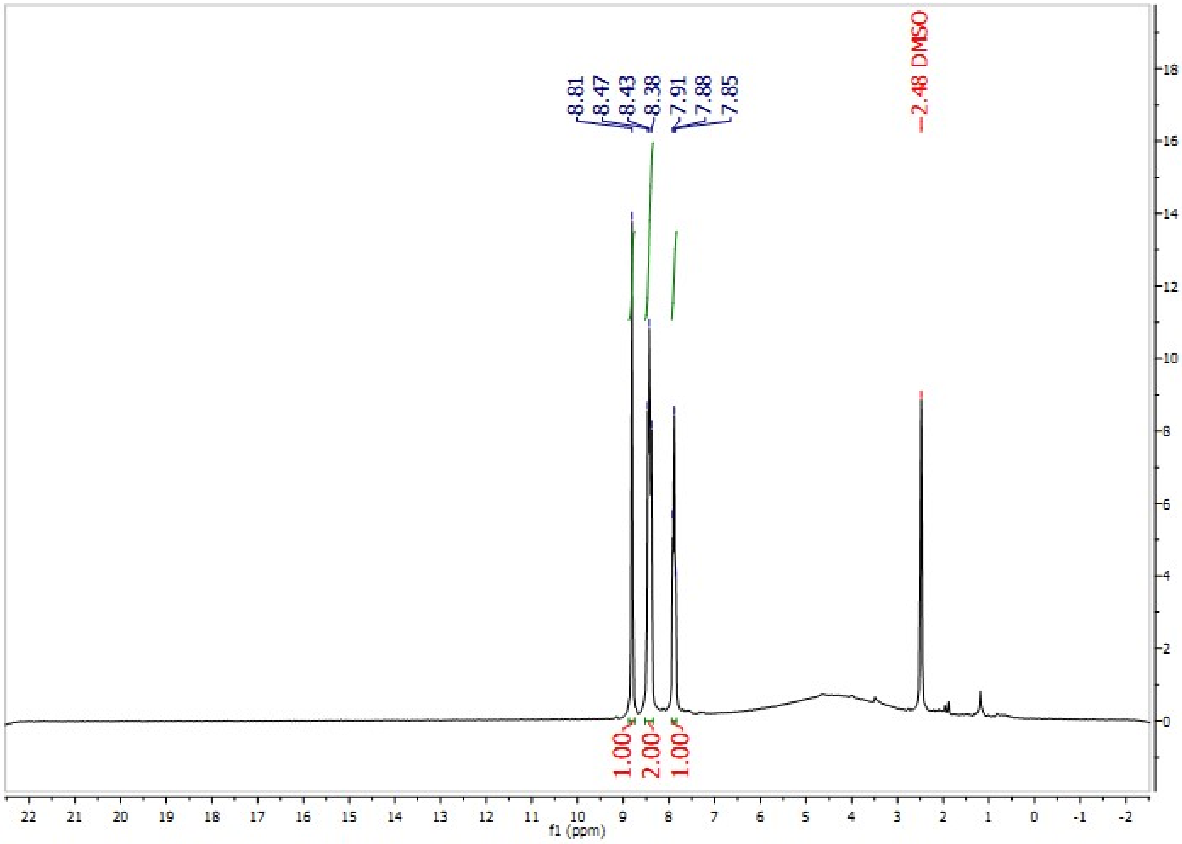

^{1}H NMR spectrum of 5-(3-nitrophenyl)-1H-tetrazole from [3+2] cycloaddition of NaN_3 with 3-nitrobenzonitrile

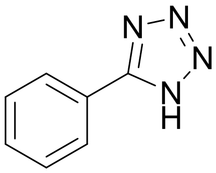

5-phenyl-1H-tetrazole

^{1}H NMR (250 MHz, DMSO): δ_H= 8.04 (m, 2H), 7.59 (m, 3H) ppm.

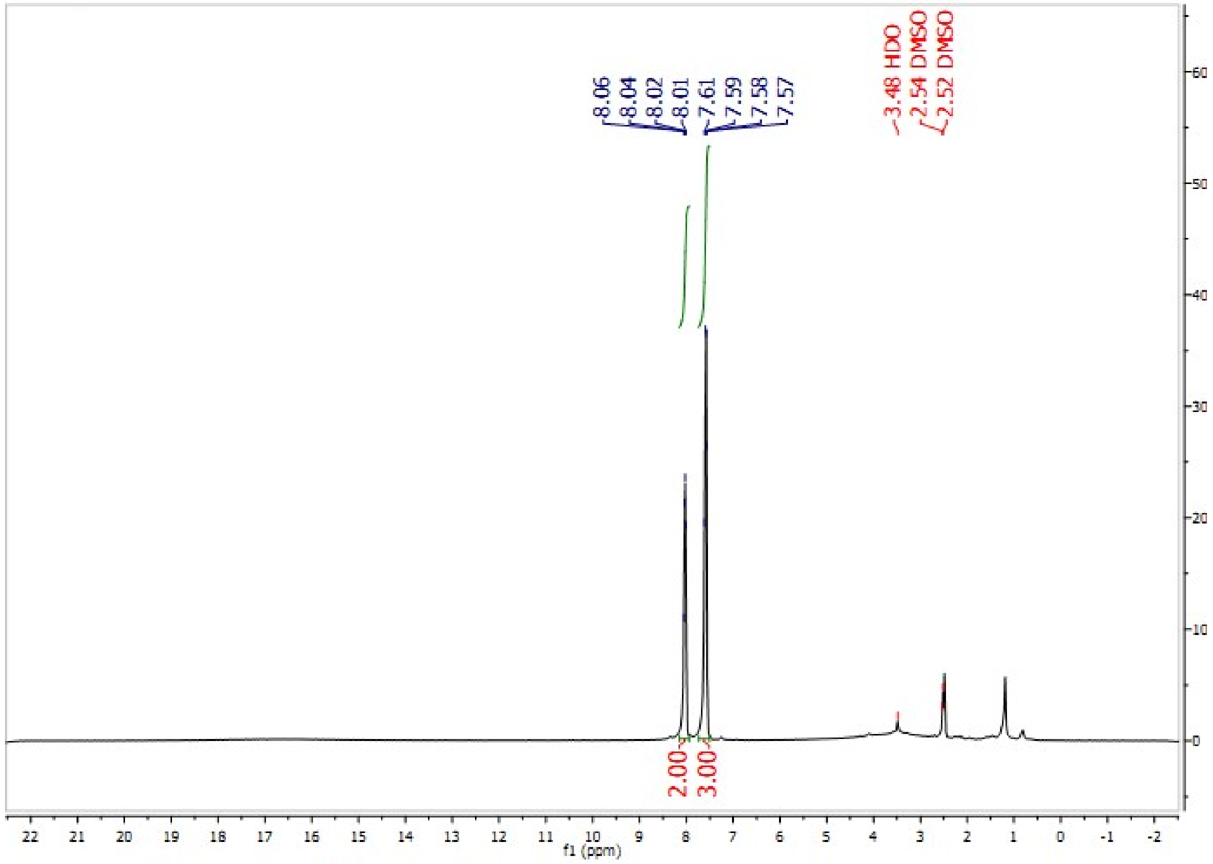

^{1}H NMR spectrum of 5-phenyl-1H-tetrazole from [3+2] cycloaddition of NaN_3 with 3-nitrobenzonitrile

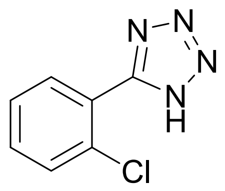

5-(2-chlorophenyl)-1H-tetrazole

^{1}H NMR (250 MHz, DMSO): δ_H= 7.79 (d, *J*= 5.90 Hz, 1H), 7.70 (d, *J*= 6.35 Hz, 1H), 7.58 (m, 2H) ppm.

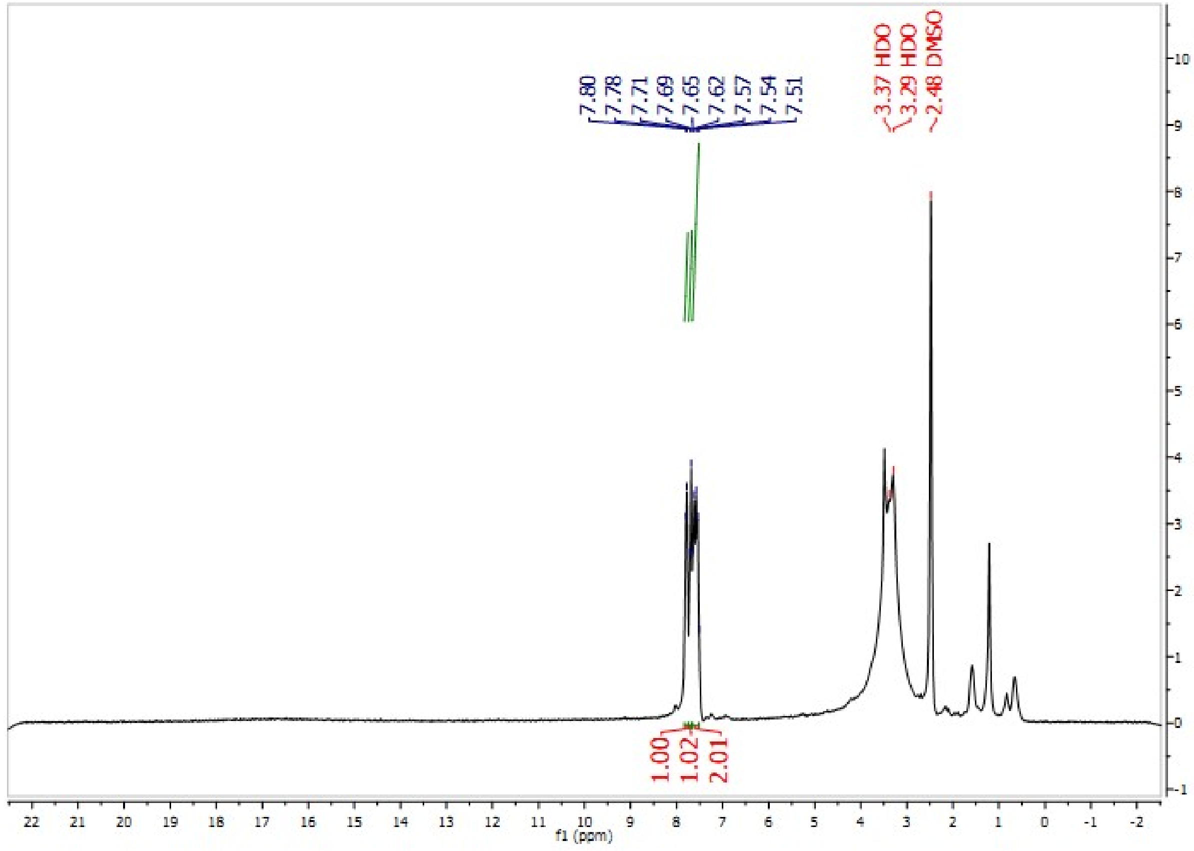

^{1}H NMR spectrum of 5-(2-chlorophenyl)-1H-tetrazole from [3+2] cycloaddition of NaN_3 with 2-chlorobenzonitrile

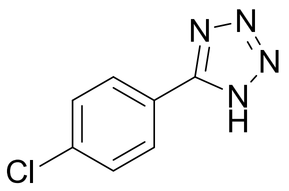

5-(4-chlorophenyl)-1H-tetrazole

^{1}H NMR (250 MHz, DMSO): δ_H= 8.03 (d, *J*= 6.65 Hz, 2H), 7.67 (d, *J*= 6.75 Hz, 2H) ppm.

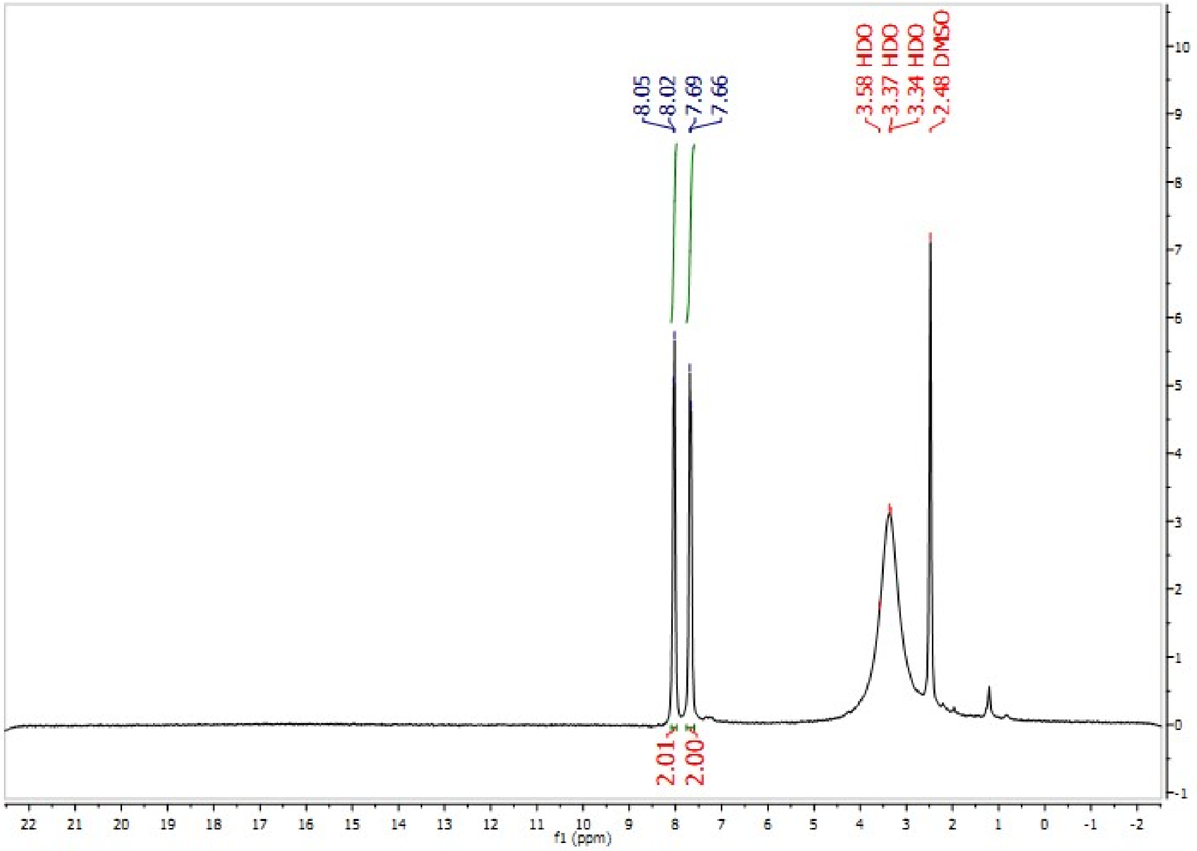

^{1}H NMR spectrum of 5-(4-chlorophenyl)-1H-tetrazole from [3+2] cycloaddition of NaN_3 with 4-chlorobenzonitrile
